# Multi-Tone Frequency Estimation Based on the All-Phase Discrete Fourier Transform and Chinese Remainder Theorem

**DOI:** 10.3390/s20185066

**Published:** 2020-09-07

**Authors:** Xiangdong Huang, Lu Cao, Wei Lu

**Affiliations:** School of Electrical and Information Engineering, Tianjin University, Tianjin 300072, China; xdhuang@tju.edu.cn (X.H.); 2018234082@tju.edu.cn (L.C.)

**Keywords:** all-phase discrete Fourier transform, Chinese remainder theorem, coprime undersampling, multi-tone frequency estimation

## Abstract

The closed-form robust Chinese Remainder Theorem (CRT) is a powerful approach to achieve single-frequency estimation from noisy undersampled waveforms. However, the difficulty of CRT-based methods’ extension into the multi-tone case lies in the fact it is complicated to explore the mapping relationship between an individual tone and its corresponding remainders. This work deals with this intractable issue by means of decomposing the desired multi-tone estimator into several single-tone estimators. Firstly, high-accuracy harmonic remainders are calculated by applying all-phase Discrete Fourier Transform (apDFT) and spectrum correction operations on the undersampled waveforms. Secondly, the aforementioned mapping relationship is built up by a novel frequency classifier which fully captures the amplitude and phase features of remainders. Finally, the frequencies are estimated one by one through directly applying the closed-form robust CRT into these remainder groups. Due to all the components (including closed-form CRT, the apDFT, the spectrum corrector and the remainder classifier) only involving slight computation complexity, the proposed scheme is of high efficiency and consumes low hardware cost. Moreover, numeral results also show that the proposed method possesses high accuracy.

## 1. Introduction

Frequency measurement is a fundamental problem in signal processing, which is widely encountered in instrumentation, digital communication, radar, etc. Numerous frequency estimation methods have been proposed, including the classical discrete Fourier transform (DFT) [1,2], the subspace-based methods [3,4], maximum likelihood [5,6], linear or nonlinear least squares [7,8]. However, as some applications work in a wider band and higher frequency, e.g., the millimeter-wave band in 5G technologies, these methods become impractical, since the realizable sampling rates of the analog to digital converters (ADCs) are limited by the Nyquist theorem. Therefore, investigations on the frequency estimation from undersampled sequences are interesting.

The Chinese Remainder Theorem (CRT) is an effective approach to resolve ambiguity related problems including the undersampling frequency estimation [9,10,11,12,13,14]. The basic idea is to reconstruct a larger number *M* from its residue set $\left\{r_{l},l=1,...,L\right\}$ modulo multiple moduli $\left\{M_{l},l=1,...,L\right\}$. Concerning the frequency estimation problem, *L* ADCs with sub-Nyquist sampling rates $\left\{M_{l},l=1,2...,L\right\}$ are employed to obtain the undersampled waveforms, and then the classical DFT is performed on the acquired waveforms to detect *L* ambiguous frequencies $\left\{{\hat{r}}_{l},l=1,2,...,L\right\}$. Moreover, compared to some searching based estimator for undersampled waveforms [9,11], the CRT-based estimator [13,15] can achieve a large reconstruction upper bound, which equals the least common multiple (lcm) of the undersampling rates $\left\{M_{l}\right\}$.

Recently, some modified versions of the CRT reconstruction algorithms have been presented for computation complexity reduction and robustness enhancement [10,11,13,14,16]. Specifically, the reduced complexity owns to the fact that CRT can work in a closed-form way rather than in a searching-based way. Besides, the robustness enhancement lies in the fact that CRT can also acquire a high reconstruction accuracy when all the remainders are erroneous, as long as these remainder errors do not exceed a quarter of the greatest common divisor (gcd) of all the moduli. Particularly, the remainders of the existing CRT-based estimators are derived from the DFT results of undersampled waveforms. Hence, if we can resort to another high-performance spectral analysis tool to replace DFT, both accuracy and anti-noise robustness will be further enhanced.

As is well known, DFT spectral analysis has two drawbacks: heavy spectrum leakage arising from data truncation and insufficient spectral resolution incurred by picket fence effect. To suppress the spectral leakage, we proposed the all-phase DFT (apDFT) spectral analysis in [17] and pointed out that apDFT spectrum’s sidelobe leakage is much slighter than DFT even when dealing with multi-tone waveforms. Moreover, as [18] pointed out, if apDFT is combined with the technique of spectrum correction, the spectral resolution can be improved significantly. This combination actually provides us a good idea to obtain higher-accuracy remainders required by CRT reconstruction.

In recent years, many endeavors have been made to generalize CRT-based estimators to multi-tone undersampled waveform cases [19,20,21]. Generally, these estimators solve this problem through the remainder redundancy coding, which actually pays the cost of heavy computation burden and sacrificing the dynamic estimation range. To break the dilemma, we develop a novel estimator combining closed-form CRT, apDFT and spectrum correction. Different from the existing estimators, our proposed estimator’s applicability in the multi-tone case is ensured by building up a mapping relationship between an individual tone and its corresponding remainders. Specifically, this mapping relationship is realized by a novel harmonic-parameter clustering method, which is closely related to apDFT and spectrum correction. With the above considerations, the proposed multi-tone estimator can be converted into multiple single-tone estimators, and thus individual tones can be retrieved one by one. Numerical results show that, our proposed estimator concurrently possesses high accuracy and large dynamic range. Moreover, our proposed estimator is applicable to the case of only 2 undersampling paths, whereas the existing multi-tone estimators cannot apply to this case.

The remaining of this paper is organized as follows. Problem Formulation of CRT-based frequency estimation is given in Section 2. In Section 3, we detail the remainder acquisition and remainder classification based on the apDFT and harmonic-parameter clustering in the proposed method. The numeral results and performance analysis are presented in Section 4. Finally, the conclusions are drawn in Section 5.

## 2. Problem Formulation

### 2.1. CRT Reconstruction Model for the Single-Tone Case

In this subsection, the CRT reconstruction model for the single-tone case is formulated. A narrow-band signal x(t) with a single tone is formulated as

(1) $$x\left(t\right)=ae^{j(2\pi f_{0}t+\theta_{0})}+w\left(t\right),$$

where *a*, $\theta_{0}$ and $f_{0}$ are the amplitude, initial phase and the frequency to be determined, respectively. w(t) is the additive white Gaussian noise with zero mean and variance $\sigma^{2}$.

The Nyquist sampling theorem requires that the sampling rate $F_{l}$ must be at least as twice as the signal frequency $f_{0}$ to avoid the ambiguity. Definitely, the ambiguity occurs in the undersampling case, i.e., $F_{l}<f_{0}$. In this case, the detected frequency $r_{l}$ can be represented as

(2) $$f_{0}=n_{l}F_{l}+r_{l},\;\;\;l=1,...,L$$

where $n_{l}$ is the folding integer, and $r_{l}$ is the ambiguous frequency which can be acquired by performing the traditional DFT on the undersampled waveforms. Equation (2) is fully in accordance with the model of closed-form robust CRT [13], providing a basis for the undersampling frequency estimation. Guided by the determination procedure in [13], x(t) needs to be discretized with *L* undersampling rates (also acting as the *L* moduli of CRT) $F_{1},...,F_{L}\ll f_{0}$. Typically, denote *N* be their greatest common divisor (i.e., the largest integer that divides each of them), namely $N=\gcd\{F_{1},\cdots ,F_{L}\}$, thus integers $\Gamma_{1},\cdots ,\Gamma_{L}$ valued with

(3) $$\Gamma_{l}=F_{l}/N,\;\;\;l=1,...,L,$$

constitute a co-prime integer set. As such, the moduli $F_{l}$ can be decided by the coprime integer set $\left\{\Gamma_{l}\right\}$ and one specified integer *N*.

Then, *L* undersampled versions of x(t) are generated as

(4) $$x_{l}\left(n\right)=ae^{j\left(2\pi \frac{f_{0}}{F_{l}}n+\theta_{0}\right)}+w\left(n\right),\;\;n=0,...,N-1.$$

Since $f_{0}\gg F_{1},...,F_{L}$, a simultaneous congruence equation set can be built up as

(5) $$\left\{\begin{array}{c} f_{0}=n_{1}F_{1}+\varepsilon_{1}F_{1}\\ \;\;\;\;\vdots \\ f_{0}=n_{L}F_{L}+\varepsilon_{L}F_{L} \end{array}\right.,$$

where $n_{1},...,n_{L}$ are unknown folding integers and $\varepsilon_{1},...,\varepsilon_{L}$ are normalized frequencies of *L* undersampled sequences. As mentioned above, the ambiguous frequencies $\left\{\varepsilon_{l}F_{l}\right\}$ (the remainders) can be approximated via the traditional DFT. Then, the frequency estimate ${\hat{f}}_{0}$ can be acquired by feeding the remainders $\left\{\varepsilon_{l}F_{l}\right\}$ and moduli $\left\{F_{l}\right\}$ into the CRT reconstruction algorithm in [13].

The analysis above gives a simple review of frequency estimation in the framework of CRT reconstruction. However, there are some open questions associated with the above method. Firstly, the robust CRT usually requires that the error in each remainder is less than one quarter of the gcd of the moduli. Under this condition, the reconstruction error can be upper bounded by the same range as that of the remainders. For another, the remainder acquisition is always achieved by the traditional *N*-point DFT, in which the normalized frequency resolution is 1/N. Accordingly, the normalized frequency $\varepsilon_{l}$ in (5) can be represented as

(6) $$\varepsilon_{l}=\left(k_{l}+\delta_{l}\right)/N,\;\;\;l=1,...,L,$$

where $k_{l}\in \left\{0,1,...,N-1\right\}$ and $\delta_{l}$ is a fractional number ranging in the interval [−0.5,0.5). Hence, the remainder $r_{l}$ can be rewritten as

(7) $$r_{l}=\varepsilon_{l}F_{l}=\left(k_{l}+\delta_{l}\right)F_{l}/N,\;\;l=1,...,L.$$

The traditional DFT allows us to obtain the integer $k_{l}$, whereas the fractional number $\delta_{l}$ (also referring to the frequency offset) tends to be erroneous due to the spectrum leakage and the picket-fence effect in the traditional DFT analysis. Therefore, the errors arising from the DFT analysis tool inevitably give rise to the errors in the reconstruction results. In this sense, it is meaningful to resort to another high-performance spectral analysis tool to replace DFT.

### 2.2. CRT Reconstruction Model for the Multi-Tone Case

For a multi-tone signal x(t) formulated as

(8) $$x\left(t\right)=\sum_{m=1}^{\rho }a_{m}e^{j(2\pi f_{m}t+\theta_{m})}+w\left(t\right).$$

Assuming that *L* undersampling rates are the same as the single-tone case, *L* discretized versions of x(t) can be represented as

(9) $$x_{l}\left(n\right)=\sum_{m=1}^{\rho }a_{m}e^{j(2\pi \frac{f_{m}}{F_{l}}n+\theta_{m})}+w\left(n\right),\;\;l=1,...,L,n=0,...,N-1.$$

Accordingly, for these tones, ρ simultaneous congruence equation sets can be built up as

(10) $$\left\{\begin{array}{c} f_{m}=n_{1,m}F_{1}+\varepsilon_{1,m}F_{1}\\ \;\;\;\;\vdots \\ f_{m}=n_{L,m}F_{L}+\varepsilon_{L,m}F_{L} \end{array}\right.,\;\;m=1,...,\rho$$

where $\varepsilon_{l,m}F_{l}$ refers to the required CRT remainder $r_{m,l}$ for the *m*-th tone at the *l*-th reconstruction path, i.e.,

(11) $$\left\{\begin{array}{c} r_{l,m}=\varepsilon_{l,m}F_{l}\\ \varepsilon_{l,m}=\left(k_{l,m}+\delta_{l,m}\right)/N \end{array}\right.,\;\;l=1,...,L,m=1,...,\rho .$$

where $k_{l,m}\in \left\{0,...,N-1\right\}$ and $\delta_{l,m}\in \left[-0.5,0.5\right)$. For any index l∈{1,...,L}, substituting (10) into (9) yields

(12) $$x_{l}\left(n\right)=\sum_{m=1}^{\rho }a_{m}e^{j(2\pi \varepsilon_{l,m}n+\theta_{m})},\;\;n=0,...,N-1.$$

To emphasize, the above multi-tone remainder acquisition is totally distinct from that of the single-tone case. In the single-tone case, there exists one peak bin of the DFT spectrum at any reconstruction path, consequently, the estimates of remainders can be obtained through collecting all the peak bins. However, in the multi-tone case, the DFT spectrum at any reconstruction path surely contains ρ peak bins and the mapping relationship between the peak bins across different paths is unknown.

We give a simple example to illustrate the unknown relationship in the multi-tone case. We assume the three numbers are {5, 23, 181}, and 181 and two moduli are {7, 9}. In this case, the two remainder sets which can be detected are {2,5,6} and {1,5}, respectively. Considering the second remainder set {1,5}, we cannot tell which element repeats twice. As for element 5, it is unclear which remainder in the first set corresponds to it. Hence, the difficulty of the CRT algorithm for multiple numbers lies in building up the mapping relationship between each number and the corresponding remainders. Specific to the frequency estimation in the multi-tone case, the difficulty lies in categorizing ρL peak bins across *L* DFT spectra into ρ remainder classes. In this way, the multi-tone frequency estimators can be decomposed into several single-tone estimators.

## 3. Proposed Method

### 3.1. All-Phase DFT Based Remainder Acquisition

In the proposed method, we combine the apDFT and spectrum correction to achieve the remainder acquisition. The combination can restrain the spectrum leakage and mitigate the fence effect in the traditional DFT, thereby improving the remainder accuracy.

In the traditional DFT, *N* samples should be collected for the *N*-point DFT, as shown in (4) and (9). Unlike the *N*-length sampling mechanism, a (2N−1)-length sequence is required in the apDFT, from which a new *N*-length sequence can be derived for *N*-point DFT.

The key idea of the apDFT is to derive a new *N*-length sequence from the sampled (2N−1)-length sequence. Without loss of generality, the multi-tone case is considered to illustrate the windowed apDFT.

Firstly, the *N*-length sequence $x_{l}\left(n\right)$ in (9) should be expanded to the (2N−1)-length one,

(13) $$x_{l}\left(n\right)=\left\{\begin{array}{cc} x_{l}\left(n\right),\;\; & -N+1\leq n\leq N-1\\ \;\;0,\;\; & \;\;\mathrm{else} \end{array}\right..$$

Given a *N*-length window function $w_{1}\left(n\right)$, e.g., Hanning window function, *N* different sequences $x_{l,q}\left(n\right),q=0,...,N-1$ with overlapped each other can be derived from the raw (2N−1)-length sequence $x_{l}\left(n\right)$. This transformation can be achieved by multiplying $x_{l}\left(n\right)$ with the shifted window function $w_{1}\left(n+q\right)$, i.e.,

(14) $$x_{l,q}\left(n\right)=x_{l}\left(n\right)w_{1}\left(n+q\right),\;\;0\leq q\leq N-1$$

where $w_{1}\left(n+q\right)$ stands for shifting $w_{1}\left(n\right)$ by *q* to the left.

It is natural to obtain a new *N*-length sequence $x_{la}\left(n\right)$ by simply averaging the corresponding elements in the sequence set $\left\{x_{l,q}\left(n\right),q=0,...,N-1\right\}$. Alternately, it can be done by weighed averaging. Typically, given another *N*-length window function $w_{2}\left(n\right)$, the weighted sequences $\left\{y_{l,q}\left(n\right),q,n=0,...,N-1\right\}$ can be formulated as follows

(15) $$y_{l,q}\left(n\right)=\sum_{r=-\infty }^{r=+\infty }x_{l,q}\left(n+rN\right)w_{2}\left(q\right),0\leq q\leq N-1,$$

where the integer *r* is utilized to perform the *N*-point cyclic shift of $x_{l,q}\left(n\right)$. Furthermore, then the *N*-length sequence $x_{la}\left(n\right)$ can be derived as

(16) $$x_{la}\left(n\right)=\left\{\begin{array}{cc} \sum_{q=0}^{N-1}y_{l,q}\left(n\right),\;\; & 0\leq n\leq N-1\\ \;\;0,\;\; & \;\;\mathrm{else} \end{array}\right..$$

The analysis above studies *N* sequences $\left\{x_{l,q}\left(n\right),q=0,...,N-1\right\}$ to determine the unique *N*-length sequence $x_{la}\left(n\right)$. Usually, the process is referred to as the all-phase preprocessing, accordingly the signal $x_{la}\left(n\right)$ is called the all-phase signal. Similarly, the all-phase DFT $X_{la}\left(k\right)$ can be calculated by performing the *N*-point DFT on $x_{la}\left(n\right)$.

Furthermore, for a given sample point, such as $x_{l}\left(0\right)$ in (13), all sequences containing $x_{l}\left(0\right)$ are derived in (14). On the contrary, the traditional DFT considers one case q=0 only. Hence, the DFT spectrum based on the all-phase signal $x_{la}\left(n\right)$ can estimate the frequency components with smaller spectrum leakage that arises from the data truncation.

From (14), it is possible to notice that x(0) exits all the possible points in the *N*-length sequence, i.e., all the possible phase, so it is referred to as the all-phase prepossessing. This also leads to the phase invariance in all-phase DFT.

**Figure 1:**
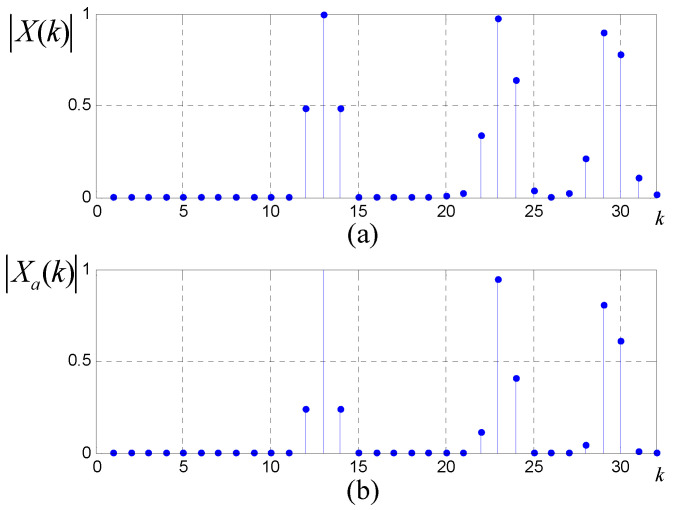
(**a**) Traditional 64-point DFT amplitude spectrum; (**b**) All-phase 64-point DFT amplitude spectrum.

**Figure 2:**
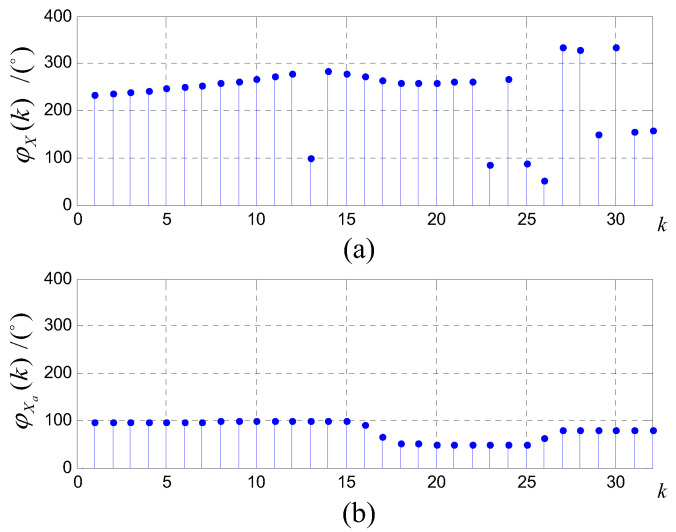
(**a**) Traditional 64-point DFT phase spectrum; (**b**) All-phase 64-point DFT phase spectrum.

In order to illustrate the superiority clearly, consider a multi-source exponential signal $x\left(n\right)\;=\;\sum_{l=0}^{\rho }e^{j(w_{l}n+\phi_{l})}$, where $N=64,w_{l}=\beta_{l}\cdot \frac{2\pi }{N}$. Assume that x(n) consists of three frequency components with $\beta_{1}=12,\beta_{2}=22.2,\beta_{3}=28.4$ respectively. The initial phases of the three components are set as $\phi_{1}=100^{\circ },\phi_{2}=50^{\circ },\phi_{3}=80^{\circ }$. The amplitude spectra and phase spectra for *N*-point windowed DFT and double-windowed all-phase DFT are shown in Figure 1 and Figure 2, respectively.

From Figure 1, we observe that the amplitude spectrum for windowed all-phase DFT has clearer peak bins and smaller side bins, verifying that the all-phase DFT can effectively restrain spectrum leakage.

From Figure 2, the phase estimates can be directly obtained around the corresponding peak bins in the phase spectrum of apDFT in Figure 2b. Especially, the phase spectrum of apDFT does not change with *k*, which differs from that of traditional DFT in Figure 2a.

Through the analysis above, all-phase DFT outperforms the traditional DFT since that the preprocessing on the (2N−1)-length sequence can significantly restrain the spectral leakage arising from the data truncation. Moreover, to reduce the errors incurred by picket effect, it is vital to adopt some correction methods to obtain accurate harmonic parameters from finite spectrum lines.

Ref. [22] pointed out that, if an exponential sequence is implemented with the Hanning windowed DFT, the ratio between the peak DFT bin $|X_{l}\left(k_{l,m}\right)|$ and its sub-peak neighbor contains the information of the frequency offset. Specifically, this amplitude ratio *v* can be calculated as

(17) $$\begin{array}{c} v=\frac{|X_{l}\left(k_{l,m}\right)|}{\max\{|X_{l}\left(k_{l,m}-1\right)|,|X_{l}\left(k_{l,m}+1\right)|\}}. \end{array}$$

In order to apply the ratio-based spectrum correction to the all-phase DFT, the amplitude ratio $v_{a}$ in our paper is specified as

(18) $$\begin{array}{c} v_{a}=\sqrt{\frac{|X_{a,l}\left(k_{l,m}\right)|}{\max\{|X_{a,l}\left(k_{l,m}-1\right)|,|X_{a,l}\left(k_{l,m}+1\right)|\}}}. \end{array}$$

On basis of [22], we can deduce the frequency offset estimate ${\hat{\delta }}_{l,m}$ as

(19) $${\hat{\delta }}_{l,m}=\left\{\begin{array}{cc} \frac{2-v_{a}}{1+v_{a}}, & \mathrm{if}\;|X_{l}\left(k_{l,m}+1\right)\left|>\right|X_{l}\left(k_{l,m}-1\right)|\\ -\frac{2-v_{a}}{1+v_{a}}, & \mathrm{else} \end{array}\right..$$

Accordingly, the normalized frequency estimate is derived as

(20) $${\hat{\varepsilon }}_{l,m}=\left(k_{l,m}+{\hat{\delta }}_{l,m}\right)/N,$$

Then, the corrected amplitude is obtained by substituting ${\hat{\varepsilon }}_{l,m}$ into the exponential term in (12)

(21) $$\begin{array}{c} {\hat{a}}_{l,m}=\pi {\hat{\delta }}_{l,m}\left(1-{\hat{\delta }}_{l,m}^{2}\right)\cdot \left|X_{l,m}\left(k_{l,m}\right)\right|/\sin\left(\pi {\hat{\delta }}_{l,m}\right), \end{array}$$

As mentioned earlier, we can directly extract the initial phase information from the phase spectrum of all-phase DFT, since that the all-phase DFT adopted in the proposed method has the excellent performance of the initial phase invariance. That means only the amplitude and frequency need be corrected via (20) and (21).

### 3.2. Harmonic Parameter Clustering Based Remainder Classification

As mentioned in Section 2.2, the main difficulty in multi-tone case lies in developing the mapping relationship between $\left\{r_{l,m}\right\}$ and $\left\{f_{m}\right\}$. Therefore, we present a harmonic parameter clustering to solve this problem.

It can be inferred from (12) that, due to multi-path undersamplings, the resultant normalized frequencies $\varepsilon_{1,m},...,\varepsilon_{L,m}$ of the *m*-th tone surely differ with each other. Nevertheless, they share a common harmonic parameter pair $\left\{a_{m},\theta_{m}\right\}$, which provides the basis for the remainder classification.

Note that the harmonic-parameter triple $\left\{{\hat{\varepsilon }}_{l,m},{\hat{a}}_{l,m},{\hat{\theta }}_{l,m}\right\}$ actually is bound together for the *m*-th tone at the *l*-th reconstruction path. To construct patterns convenient for further remainder clustering, the following vector quantities need to be built up as:

(22) $${\hat{z}}_{l,m}={\hat{a}}_{l,m}e^{j{\hat{\theta }}_{l,m}},\;\;\;\;l=1,...,L,\;\;m=1,...,\rho .$$

For any individual pattern of the *m*-th tone at the *l*-th path, we might as well select the pattern ${\hat{z}}_{1,m}$ of the 1-st path as the reference. Then, its clustering indicators $c_{l,m}$ can be determined by finding the closest distance among ρ patterns ${\hat{z}}_{l,1},...,{\hat{z}}_{l,\rho }$, i.e.,

(23) $$c_{l,m}=\arg\min_{m^{\prime }=1,...,\rho }\left\|{\hat{z}}_{1,m}-{\hat{z}}_{l,m^{\prime }}\right\|.$$

In this way, altogether ρL indicators $c_{l,m},m=1,...,\rho ,l=1,...,L,$ can be collected, besides that the reference indicators $c_{1,m}=1,m=1,...,\rho$.

Particularly, for the two-path case (i.e., L=2), two indicator-involved remainders $r_{1,m},r_{2,c_{2,m}}$ can be determined via the above harmonic-parameter clustering operations. Following this, feeding the moduli $F_{1},F_{2}$ and $r_{1,m},r_{2,c_{2,m}}$ into the procedure of the closed-form robust CRT in [13] yields the final estimate ${\hat{f}}_{m},m=1,...,\rho$.

### 3.3. Determination Procedure of Multi-Tone Frequency Based on apDFT Analysis and CRT

The proposed method for multi-tone frequency estimation can be summarized as follows (Algorithm 1).

**Algorithm 1:** The optimized discrete spectral analysis (ODSA).
**Input**: *L* coprime integers $\Gamma_{1},\cdots ,\Gamma_{L}$, the constant *N*;**Output**: Frequency estimates ${\hat{f}}_{m},m=1,...,\rho$;
Acquire (2N−1)-length sequences $x_{l}\left(n\right),n=-N+1,...,N-1$ by undersampling the signal x(t) at the sampling rates $F_{l}=\Gamma_{l}N,l=1,...,L$ respectively.At any *l*-th path, preprocess the acquired sequences $x_{l}\left(n\right)$ according to (14)–(16), from which the *N*-length all-phase sequence $x_{la}\left(n\right),n=0,...,N-1$ can be obtained.Implement *N*-point DFT on sequences $x_{1a}\left(n\right),...,x_{La}\left(n\right)$ to acquire the all-phase DFT results $X_{la}\left(k\right),...,X_{La}\left(k\right),k=0,...,N-1$. For any *l*-th path (l=1,...,L), record the peak DFT bin indices $k_{l,m},\;m=1,...,,\rho$.For each DFT bin, implement Spectrum Correction through (18)–(21) to acquire ρL harmonic parameter triples $\left\{{\hat{a}}_{l,m},{\hat{\theta }}_{l,m},{\hat{\varepsilon }}_{l,m}\right\}$, from which ρL vector quantities ${\hat{z}}_{l,m}$ can be constructed in terms of (22).For any individual ${\hat{z}}_{1,m}$ of the *m*-th tone at the 1st path (m=1,...,ρ), find out the peak indicator $c_{l,m}$ amongst $\left\{{\hat{z}}_{l,1},...,{\hat{z}}_{l,\rho }\right\}$ at the *l*-th path (l=2,...,L) according to the principle in (23).For m=1,...,ρ, substituting the remainder set $\left\{{\tilde{r}}_{1,m},...{\tilde{r}}_{l,m}\right\}$, the gcd *N*, the moduli $\left\{F_{1},...,F_{L}\right\}$ into the CRT reconstruction procedure [13] yields the final frequency estimate ${\hat{f}}_{m}$ of the *m*-th tone.


## 4. Simulation Results and Discussion

In this section, the simulation is carried in MATLAB R2016b, with an Intel Core i5 2.60 GHz. To emphasize, the existing CRT-based multi-tone frequency determination approaches [19,20,21] cannot apply to the case that the reconstruction path number *L* is smaller than the component number ρ. Therefore, this section will first verify the feasibility of the case L<ρ, meaning that there are more frequency components to be estimated than the data acquisition paths. As Ref. [22] pointed out, the Hanning Window is adopted to guarantee the applicability of spectrum correction.

### 4.1. Procedure Demonstration

For the case of L=2 and ρ=4, the corresponding frequencies, amplitudes and initial phases of the four components are listed in Table 1. To validate the proposed method in high-frequency scenarios, four tones with high frequencies (up to the GHz level) are considered. The input parameters are set as follows: the co-prime integers $\Gamma_{1}=3301,\Gamma_{2}=3307$, the gcd *N* = 512. In terms of (3), two ADC sampling rates $F_{1}=N\Gamma_{1}=1.690112\times 10^{6}$ samples/s and $F_{2}=N\Gamma_{2}=1.693184\times 10^{6}$ samples/s, much lower than the signal frequencies $f_{1},...,f_{4}$ listed in Table 1. The noise w(t) in is additive white Gaussian noise with mean zero and variance $\sigma_{n}^{2}$. In this paper, the SNR is defined as

$$\mathrm{SNR}=10\mathrm{lg}\frac{\sigma_{s}^{2}}{\sigma_{n}^{2}}$$

where $\sigma_{s}^{2}$ indicates the signal mean power. In this simulation, the SNR is specifies as 13 dB.

Through undersampling and all-phase preprocessing (Hanning double-window is adopted) in Step 1, Step 2, two *N*-length all-phase sequences $x_{1a}\left(n\right),x_{2a}\left(n\right)$ can be generated.

**Figure 3:**
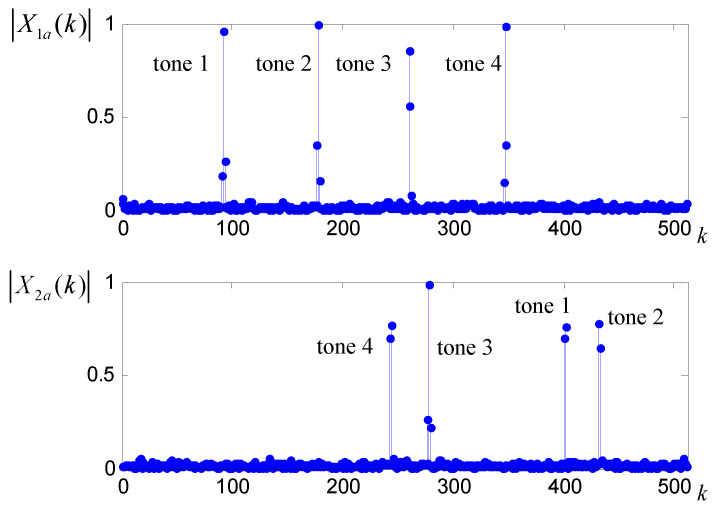
**The upper pane** shows the windowed apDFT amplitude spectrum of the undersampled sequence *x_1a_(n)*. **The bottom pane** shows that of the undersampled sequence *x_2a_(n)*.

Following Step 3, two apDFT spectra $X_{1a}\left(k\right)$, $X_{2a}\left(k\right)$ are acquired and illustrated in Figure 3, from which two sets of peak indices are recorded as $\left\{k_{1,1},...,k_{1,4}\right\}=\left\{92,178,261,347\right\}$, $\left\{k_{2,1},...,k_{2,4}\right\}=\left\{244,278,402,432\right\}$.

**Figure 4:**
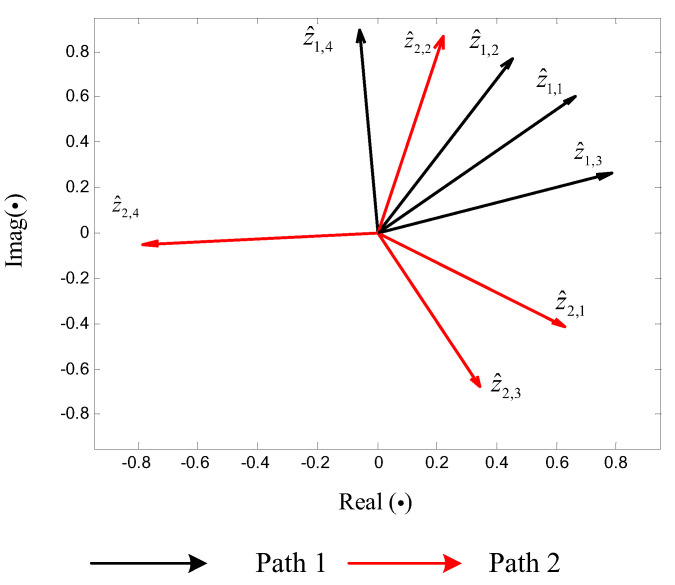
Clustering of uncorrected vector quantities.

**Figure 5:**
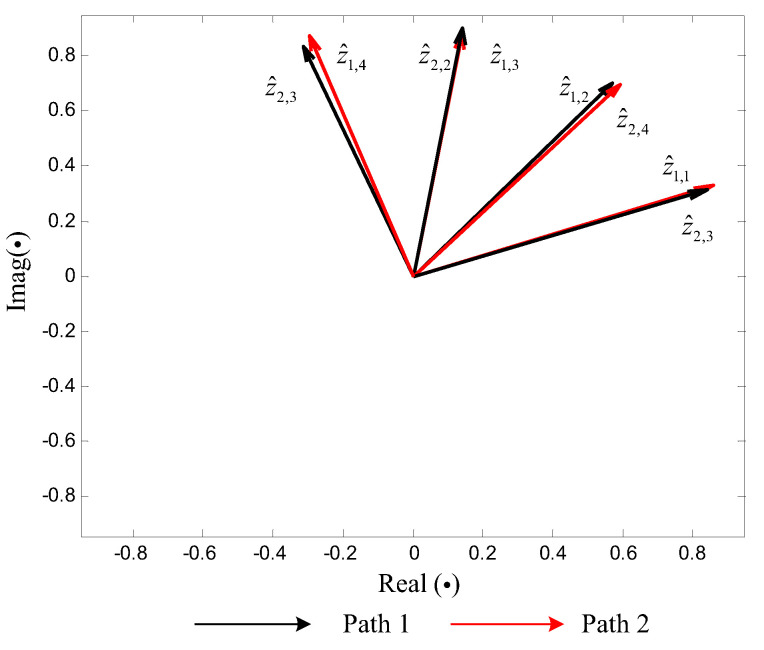
Clustering of corrected vector quantities ${\hat{z}}_{l,m}$.

Following Step 4, through applying ratio-based spectrum correction on these peak DFT bins, the ρL=8 harmonic parameter triples are determined. With that, the vector quantities and remainder information $\{{\hat{z}}_{l,m},{\hat{\varepsilon }}_{l,m}\},l=1,2,m=1,...,4$ can be generated, which are listed in Table 2. As a comparison, the uncorrected vector quantities (generated from the DFT spectra directly) and these corrected vector quantities ${\hat{z}}_{l,m}$ are illustrated in Figure 4 and Figure 5, respectively. It becomes clear that the ρL=8 vector quantities in Figure 5 can be intuitively divided into four sets, which correspond to the four tones, respectively, whereas the uncorrected patterns in Figure 4 appear chaotic.

Following Step 5, in terms of (23), we can acquire two sets of indicators $\left\{c_{1,1},...,c_{1,4}\right\}=\left\{1,2,3,4\right\}$, $\left\{c_{2,1},...,c_{2,4}\right\}=\left\{3,4,2,1\right\}$. In other words, 8 vector quantities are clustered into ρ=4 pairs as $\left\{{\hat{z}}_{1,1},{\hat{z}}_{2,3}\right\}$, $\left\{{\hat{z}}_{1,2},{\hat{z}}_{2,4}\right\}$, $\left\{{\hat{z}}_{1,3},{\hat{z}}_{2,2}\right\}$, $\left\{{\hat{z}}_{1,4},{\hat{z}}_{2,1}\right\}$, as Figure 5 depicts. Further, in terms of ${\tilde{r}}_{l,m}\;=\;{\hat{\varepsilon }}_{l,c_{l,m}}F_{l},\;l=1,2$, ρ=4 remainder sets $\left\{{\tilde{r}}_{1,m},{\tilde{r}}_{2,m}\right\}$ are acquired and listed in Table 3.

Following Step 6, successively substituting these 4 remainder sets, the gcd N=512 and the moduli $\left\{F_{1},F_{2}\right\}$ into the closed-form CRT reconstruction procedure [13] yields the final frequency estimates ${\hat{f}}_{1},...,{\hat{f}}_{4}$ listed in Table 4, which reflect that the errors are almost negligible.

In this simulation, we present the step-by-step frequency determination procedure based on the given parameters. For the case of more frequency components than the data acquisition paths, the effectiveness of the proposed method has been verified. In addition, the results also verify the applicability to the high-frequency scenario.

### 4.2. Performance Analysis on the All-Phase DFT and Traditional DFT

By following the above steps, the multiple frequency estimates can be obtained in the two-path case. Since that the traditional DFT is the most commonly used technique for remainder acquisition, we conduct the experiment to verify the superiority of the apDFT over the traditional DFT in the remainder acquisition.

**Figure 6:**
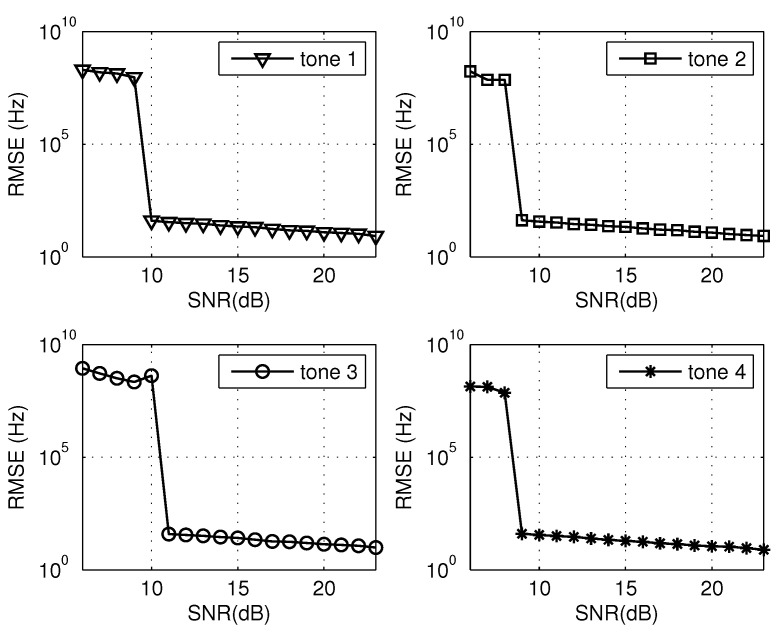
RMSE results of four tones utilizing the all-phase DFT.

**Figure 7:**
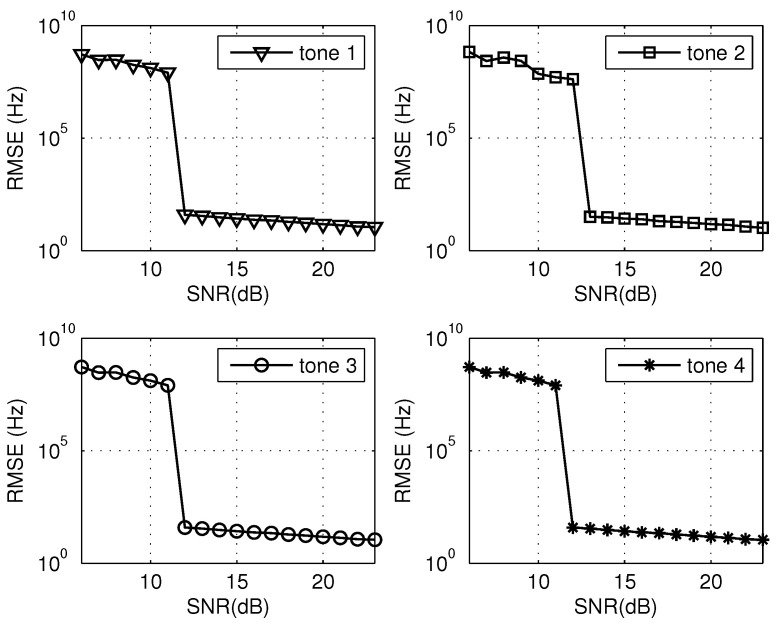
RMSE results of four tones utilizing the traditional DFT.

Based on the same multi-tone signal given in Section 4.1, the estimation accuracy of the proposed approach across different tones versus SNRs ranging from 6 dB to 23 dB is studied. Herein, the significance of SNR is identical with that in Section 4.1. The root-mean-square error (RMSE) is defined as $\mathrm{RMSE}=\sqrt{1/Q\sum_{i=1}^{Q}{\left({\hat{f}}_{mi}-f_{m}\right)}^{2}}$, where the superscript *i* refers to the *i*-th trial and *Q* denotes the number of Monte Carlo tests. For each SNR case, 500 Monte Carlo trials were conducted. The RMSE curves of four tones are shown in Figure 6. To ensure the comparability, another experiment was conducted under the same condition except that the remainder acquisition was achieved by the traditional DFT. Similarly, the RMSE results of four tones are shown in Figure 7.

As Figure 6 and Figure 7 depict that, for any tone, there is a SNR threshold under which the estimation error abruptly increases. For the region where the SNR is above the threshold, the relative errors corresponding to any tone are below the level $100/10^{9}\times 100\%=0.00001\%$. The result matches perfectly with that of the single trial in Section 4.1.

As a comparison, for any tone, the threshold value of the proposed method (utilizing the all-phase DFT) in Figure 6 is smaller than that utilizing the traditional DFT in Figure 7. The simulation confirms that the apDFT can result in the improvement of anti-noise robustness compared with the traditional DFT. The improvement in anti-noise is due to the property of restraining spectral leakage of apDFT, which can detect remainders with higher accuracy. Essentially, it can be attributed to the all phase preprocessing mechanism.

### 4.3. Performance Analysis on Different Number of Data Acquisition Path

In this simulation, some simulation results are presented to investigate the estimation performance in different data acquisition number. For simplicity and effectiveness, the overall RMSE of four tones is used to assess the frequency estimation performance. Herein, the method utilizing the traditional DFT (as mentioned in Section 4.2) is also considered as a reference.

**Figure 8:**
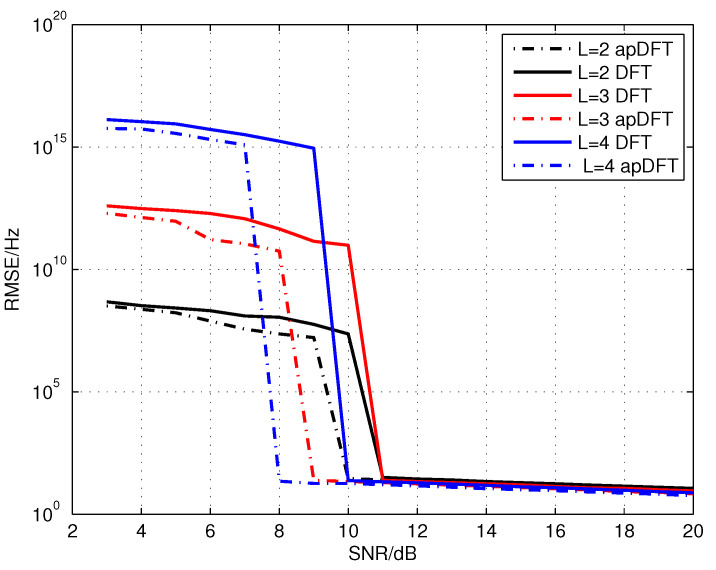
RMSE results of frequency estimation under different number of data acquisition path.

Figure 8 illustrates the RMSE results under the channel number L=2,3,4 based on the model parameterization in Section 4.1. For each channel number, the apDFT-based method has a better performance. This corresponds to the results in Figure 6 and Figure 7. For another, as to the proposed method, the more data acquisition paths, the smaller the value of threshold is. To some extent, the robustness can be enhanced by increasing channel numbers, whereas the enhancement cannot be ensured when the peak bins are contaminated by the heavy noise. In addition, it is interesting to observe that, in the low SNR region, the error magnitude associated with more data acquisition paths like the channel number L=4 is significantly higher. This occurs when the clustering results are invalid due to heavy noise contamination, and then more cumulative errors are generated with the increasing channel number.

### 4.4. Analysis of Computation Complexity

In the proposed method, the multi-tone frequency estimation is achieved by incorporating the apDFT, the harmonic-parameter clustering and the closed-form CRT reconstruction algorithm. Note that the CRT reconstruction algorithm adopted works in a closed-form way. Furthermore, the spectrum correction and the harmonic-parameter clustering only involve in simple algebra calculations. Therefore, the computation complexity of the proposed estimator mainly depends on the *N*-point apDFT in *L* data acquisition paths, i.e., $O(N/2\log_{2}N)$. Compared with the existing CRT-based estimators, the proposed method works well even when the data acquisition path number equals two and no searching step is required. Obviously, no heavy computation burden is required in the proposed method.

## 5. Conclusions

In this paper, we propose a multi-tone frequency estimator from undersampled waveforms, which incorporates the closed-form CRT, the apDFT spectral analysis, the spectrum correction, and the harmonic-parameter clustering. This organic technique combination allows that the multi-tone estimator is decomposed into multiple single-tone estimators. Thus these tones can be recovered one by one. Two remarkable merits should be emphasized.

On one hand, different from the existing CRT-based multi-tone estimators with remainder redundancy coding, the proposed estimator can still work well even if the reconstruction path number L=2, which actually greatly reduces the hardware cost. On the other hand, due to the utilization of apDFT, which yields excellent suppressing effect of spectral leakage and noise, our proposed estimator can acquire a higher anti-noise robustness than the DFT case.

The above two merits give the proposed estimator with vast potentials in high-frequency measurement related applications such as radar, future beyond 5G communications.

## Figures and Tables

**Table 1:** Harmonic parameters of the multi-tone signal.

Tones	$f_{m}$ (Hz)	$A_{m}$	$\theta_{m}$
$s_{1}\left(t\right)$	$1.3\times 10^{9}$	1	$20^{\circ }$
$s_{2}\left(t\right)$	$1.4\times 10^{9}$	1	$50^{\circ }$
$s_{3}\left(t\right)$	$0.9\times 10^{9}$	1	$80^{\circ }$
$s_{4}\left(t\right)$	$1.12\times 10^{9}$	1	$110^{\circ }$

**Table 2:** The vector quantities and normalized frequencies of two undersampled sequences in Step 4.

		m=1	m=2	m=3	m=4
*l* = 1	${\hat{z}}_{1,m}$	0.99$e^{j22.5^{\circ }}$	0.98$e^{j51.2^{\circ }}$	1.03$e^{j81.9^{\circ }}$	1.00$e^{j107.1^{\circ }}$
	${\hat{\varepsilon }}_{1,m}$	0.1798	0.3475	0.5090	0.6780
*l* = 2	${\hat{z}}_{2,m}$	1.00$e^{j107.7^{\circ }}$	1.01$e^{j83.5^{\circ }}$	1.01$e^{j18.2^{\circ }}$	1.01$e^{j49.2^{\circ }}$
	${\hat{\varepsilon }}_{2,m}$	0.4757	0.5429	0.7842	0.8446

**Table 3:** The classified CRT remainders and normalized frequencies in Step 5.

	m=1	m=2	m=3	m=4
${\hat{\varepsilon }}_{1,c_{1,m}}$	0.1798	0.3475	0.5090	0.6780
${\hat{\varepsilon }}_{2,c_{2,m}}$	0.7842	0.8446	0.5429	0.4757
${\tilde{r}}_{1,m}$	$0.3038\times 10^{6}$	$0.5873\times 10^{6}$	$0.8604\times 10^{6}$	$1.1459\times 10^{6}$
${\tilde{r}}_{2,m}$	$0.8054\times 10^{6}$	$0.9193\times 10^{6}$	$1.3279\times 10^{6}$	$1.4300\times 10^{6}$

**Table 4:** Final results of frequency recovery.

	m=1	m=2	m=3	m=4
$f_{m}$ (Hz)	$1.3\times 10^{9}$	$1.4\times 10^{9}$	$0.9\times 10^{9}$	$1.12\times 10^{9}$
${\hat{f}}_{m}$ (Hz)	$1.2999\times 10^{9}$	$1.3999\times 10^{9}$	$0.8999\times 10^{9}$	$1.1200\times 10^{9}$
